# “There's just too many”: The construction of immigration as a social problem

**DOI:** 10.1111/1468-4446.12933

**Published:** 2022-03-01

**Authors:** James Pattison

**Affiliations:** ^1^ Department of Sociology University of Manchester Manchester UK; ^2^ The School of Social and Political Sciences University of Lincoln Lincoln UK

**Keywords:** class, deindustrialisation, ethnography, migration, place

## Abstract

This article presents findings collected in 2016–2017 from a multi‐method ethnographic study of Shirebrook, Derbyshire in the English East Midlands, examining the narratives used by the local authority (LA) and local residents that construct immigration as a social problem. In doing so, it contributes to the literature on race and migration by extending analysis beyond metropolitan localities with long histories of multi‐ethnic settlement, to consider a relatively small, peripheral former colliery town. The paper demonstrates how migration is framed as a social problem by central government funding streams with consequences for localities, and the influence this has on local narratives of social change. The construction of immigration as a social problem is rooted in the constraints of austerity and longer‐term processes of deindustrialization and economic restructuring, with representations and understandings of place being constitutive of anti‐immigrant sentiment. This article deepens our understanding of responses to immigration in the UK, and has broader implications for understanding the relationship between place, state polices and local narratives.

## 1 INTRODUCTION

This article examines the narratives used by a local authority (LA) and local residents that construct immigration as a social problem. Britain's post‐industrial towns have been at the center of recent debates over emergent right‐wing populism, represented by a number of key events, most recently including Brexit and the collapse of the so‐called “red wall”^1^ in the 2019 general election. Yet post‐industrial towns remain relatively under‐researched in the context of race and migration. This research extends analysis of race and migration beyond large urban areas with long histories of diversity, to consider a relatively small, and predominantly white, former colliery town that falls “in‐between” an urban/rural binary (Burdsey, 2016; Nayak, 2010). The findings illustrate how the construction of immigration as a social problem is rooted in constraints of austerity and longer‐term processes of deindustrialization and economic restructuring, representations and understandings of place, and how central government funding streams encourage local authorities to frame social problems in particular ways. The article contributes to our understanding of how immigration is constructed as a problem, exploring how this construction works through place at various scales and how place itself is constitutive of anti‐immigrant sentiment. In addition to contributing to how we understand responses to immigration in the UK, there are broader implications for understanding the relationship between state policies and local narratives about migration.

The following section reviews literature on race and migration, emphasizing the significance of place and attentiveness to geographical contexts for understandings of situated encounters with racialized others. The fieldwork site, research context and methods are then discussed. Three analytical sections then follow: The first examines how migrant numbers are used to construct immigration as a social problem, foregrounding the Local Strategic Partnership's *Building Resilience* program (2017) and how this is shaped by the economic context and government funding, before documenting how the numbers game is played by the LA, and how this features in residents' interview testimonies. The second section investigates the symbolic representations of Shirebrook as both urban and rural and the variable mobilizations of these landscape representations to construct immigration as problematic and legitimize its opposition. The final section examines access to resources, in particular the LA's role in defining immigration as the cause for declining services, constructing it as *the* legitimate social problem affecting the town.

## 2 PLACE, RACE AND MIGRATION

Focused on the “superdiversity” (Vertovec, 2007) and “urban multiculture” (Jones et al., 2015) of major urban settings, post‐industrial towns have been largely absent from race and migration scholarship. The applicability of findings in major cities to places that are more “representative of where most of the British population is likely to be living” (Valluvan, 2019, p. 41) remains largely unknown. Changes in migration flows to areas with comparatively less historical diversity has increased this need to look beyond the city (Burdsey, 2016; Vertovec, 2007). For instance, migrants from European Accession^2^ nations tend to be drawn to meet labor demands created by low‐wage economies in demographically aging and depopulating post‐industrial towns. Emerging sociological research has begun to focus on arguably more mundane and peripheral locations, such as Peterborough (Rogaly, 2020); Stoke‐on‐Trent (Burnett, 2011); northern English “mill towns” (Miah et al., 2020; Rhodes, 2011; Thomas et al., 2018); the English seaside (Burdsey, 2016; Grillo, 2005); and rural locations (Neal, 2002; Tyler, 2012). However, there are few studies of race and migration within the distinct socio‐spatial context of deindustrialising colliery towns.

The urgency of extending sociological analysis of race and migration beyond metropolitan areas has been initiated by several key moments. First, to make sense of riots in several Northern English mill towns in 2001, and the electoral success of the British National Party in the early 2000s (Miah et al., 2020; Rhodes, 2011). Second, in the wake of Brexit and the collapse of the so‐called “red wall” in the 2019 general election. So‐called “left behind”^3^ post‐industrial towns were argued to be pivotal to this political realignment (Goodwin & Heath, 2016; McKenzie, 2017); however, closer analysis of the Brexit vote suggests a more complex picture (Chan et al., 2020; Dorling, 2016). The centrality of the “left behind” to emergent forms of right‐wing populism extends beyond the UK and is discussed in the context of mainland Europe (Scheiring, 2020) and the United States (Hochschild, 2018).

Attention to place is vital to understand situated encounters with racialized others (Nayak, 2010; Neal, 2002; Parker & Karner, 2010). Burdsey's (2016) conceptualization of the seaside is instructive for making sense of place's significance in relation to former coalfields. First, we are encouraged to think beyond the urban/rural binary to consider geographical “‘third spaces’ … that fall ‘in‐between’ the city and the countryside” (Burdsey, 2016, p. 88; Nayak, 2010). Burdsey (2016) notes how the predominance of positioning racialized minorities within urban settings reifies an association between people and place, denying their existence beyond deprived inner‐city locations. While the countryside is imagined as a timeless white space, and site of safety and retreat from urban multiculture (Neal, 2002). This illustrates “how race is spatialised and space is racialised” (Burdsey, 2016, p. 83), implicit in the “left behind” characterization of post‐industrial towns which has come to signify a narrowly defined *white* (British) working‐class (Bhambra, 2017; Rhodes et al., 2019).

Former mining communities provide a distinct socio‐spatial location to explore race and migration. Although different to the seaside, coalfield communities can also be thought of as “in‐between” urban and rural and were characterized as such in regeneration literature that highlighted how urban deprivation and decay coincides with rural isolation (Townsend & Hudson, 2005). Beynon and Hudson (2021) also illustrate the coalfields' in‐betweenness through their development in the countryside away from the urban centers typically associated with industrialization. Industrial decline meant that many coalfield communities were officially (re)declared as rural without reverting “to an agrarian past” and so do not fit “easily into any map” (Beynon & Hudson, 2021, p. 236). Sociological research on coalfields emphasized physical isolation, mono‐industrial character, and strong forms of social solidarity mediated and strengthened through networks associated with the colliery, trade union and welfare institutes (Bulmer, 1975). This characterization has been justifiably criticized for homogenizing mining communities (Strangleman, 2018), while others have made a distinction between tight‐knit, single industry and isolated *miners'* towns, with more economically and socially diverse *mining* towns (Gilbert, 1995). Nevertheless, the physical isolation and prior dominance of a single industry is useful for contemporary understanding of many former mining communities.

The status of coal mining in the national imagination has fluctuated. Like the seaside (Burdsey, 2016) and English countryside (Bonnett, 1998; Neal, 2002), mining communities were periodically construed as sites of national pride and identity, providing the fuel to power industrial revolution and empire (Ebke, 2018). As such, the coalfields have assumed a central role in narratives of the nation, which fixes “particular types of racialised bodies within and outside it” (Burdsey, 2016, p. 19). But this elevated status was contingent. Industrialization as it coincides with the urbanization of colliery towns was also associated with racial degeneration (Bonnett, 1998) and historically miners and mining communities were constructed as a “race apart”. From “the earliest days of industrial mining, metaphors of darkness, difference and racial otherness spilled out from below ground onto the face of the earth, marking off mining settlements from the rest of the nation” (Gilbert, 1995, p. 48). The status of mining communities waned after the 1984–1985 miners' strike from the “c*ause célèbre* of the liberal intelligentsia” (Bright, 2011, p. 67) to the “enemy within”, as labeled by then Prime Minister, Margaret Thatcher. After the industry ended, this status has transformed into the “left‐behind”, providing the basis for nostalgic narratives of “‘coal nationalism’ where coal is [again] turned into a key symbol of security … in relation to threatening outsiders” (Thorleifsson, 2016, p. 566). As such, coal mining communities have at various times been seen as deserving or undeserving constituents of the nation (Shilliam, 2018).

Walkerdine and Jimenez (2012, p. 55) argue that when small communities grow around an industry a strong sense of “communal beingness” develops through shared histories of social change, solidarity, trade unionism, and struggle. This has the capacity to produce a fear of outsiders and reluctance to move away. However, this potentially characterizes post‐industrial communities as static, “narrow and parochial” (Strangleman et al., 1999, p. 1.1). Migration within the UK and Ireland was a feature of mining communities, with families moving from declining coalfields to more productive collieries (Phillips, 2018), and British miners migrated to coalfields across the globe (Knotter, 2015). Gilbert (1995) illustrates this complexity, emphasizing that mining communities' identities derive from interactions with the outside world through migration, and the influence of education and popular culture. Migration included the introduction of European Voluntary Workers from Eastern Europe and Italy to the mining industry post‐World War II, despite resistance from trade unions (Catterall & Gildart, 2005; McDowell, 2009). Other work has indicated diversity in the coalfields, such as a recent heritage project highlighting the role of Black and African‐Caribbean miners in the UK coal industry (www.blackcoalminers.com). Tyler's (2012) ethnographic research in a former coal mining town in Leicestershire also demonstrates younger residents questioning racism and the colonial worldview of some of the town's older residents. This further problematizes the image of the narrow and parochial former coal mining community, revealing the potential for conviviality to exist alongside conflict (Karner & Parker, 2011).

Economic context is key to understanding migration in peripheral post‐industrial towns. Labor demand has underpinned changing migration patterns through job growth in insecure low‐waged work in former industrial areas, which relies disproportionately on migrant labor (Beatty & Fothergill, 2017). This is compounded by austerity, which has severely reduced welfare and public services, impacting Britain's older industrial areas particularly hard (Beatty & Fothergill, 2017). Hansen's (2021) recent research in Sweden challenges the conventional assumption that low‐earning migrants and refugees are a fiscal burden and threat to the welfare state. Rather, Hansen (2021) demonstrates that, rather than austerity, proactive and significant increases in government spending in demographically aging and depopulating localities whilst directing migrants into those areas, leads to economic growth and increased tax revenues. But also leads to *real* resources in the form of new inhabitants and labor to offset the impact of aging and population decline. However, increased migration into an impoverished context contributes to a sense of competition between settled communities and new migrants over few resources (Lloyd et al., 2021; Miah et al., 2020). The matter is made more complex when the competition is also presented in racialized terms.

However, economic context is inadequate to make sense of responses to migration on its own. Bhambra (2017) criticizes the “left behind” conceptualization in analysis of the Brexit vote for its “methodological whiteness” which obscures the racialized narratives operating in emergent right‐wing populism, as well as the impact economic restructuring has on minorities and migrants (see also Miah et al., 2020; Rhodes et al., 2019). Lloyd et al.'s (2021, p. 8) research on migration in an area still contending with the legacy of deindustrialization illustrates the fractious ways immigration is responded to in the context of perceived decline and socio‐economic disadvantage. However, they offer no explanation as to why some “white” working‐class residents misidentify migrants as ‘the true cause of anxiety and socio‐economic status’, questioning whether identifying an “object of fear” is in fact racist (Lloyd et al., 2021, pp. 16–17). As Lentin (2020, p. 57) argues, the “adjudication of whether a statement, an action, or a process is racist … eschews engagement with what race *does* as a discursive and performative regime”. The reduction of race to questions of individual morality therefore “bypasses a more nuanced analysis of the landscape in which these events play out” (Lentin, 2020, p. 58). This is significant in the context of widespread popular and political anti‐immigrant sentiment, particularly in the lead up to the EU referendum (Mondon & Winter, 2020; Moore & Ramsay, 2017; Virdee & McGeever, 2018). Anderson (2017, p. 1533) contends that Brexit resulted from ‘multiple policies that disenfranchised and impoverished millions of people on the one hand, and years of setting up “migrants” as the reason for the lack of jobs, low wages, [and] poor public services’. This leads us to question what role the state and media play in constructing racialized interpretations of social problems whilst simultaneously obscuring alternative accounts.

Media representations of recent East European migrants use a number of well‐worn racialising frames to construct them as threatening and “not white” (Fox et al., 2012; Rasinger, 2010), contributing to the shaping of public perceptions of migration (Blinder & Allen, 2015). Drawing on Bourdieu's (1991) theory of symbolic power, Tissot (2018, p. 151) demonstrates the “social production of social problems” via state policies aimed at strengthening community cohesion and the categorization of particular French urban neighborhoods as “sensitive areas”. Implicit in these policies was a racialising frame that constructs immigrants as “people who created social ills” rather than face them (Tissot, 2018, p. 153). Therefore, the state plays a key role in defining problem populations and constructing which social problems are to be perceived as legitimate. A second consequence of this policy framing is that ‘problems’ are framed as local level cohesion issues divorced from broader systemic inequalities. In the UK, community cohesion and integration policies are similarly active in the construction of social problems, as well as place‐images including categorizing so‐called “failed spaces of multiculturalism” or identifying the “white” working‐class as a threat to national cohesion (Jones, 2015; Miah et al., 2020; Thomas et al., 2018). In their extensive research in the satellite “mill towns” around Manchester and Leeds, Miah et al. (2020, p. 246) note that community cohesion policy portrays “both ‘race’ and the agency of racialised ‘communities’ as being causal to the (real and substantial) social problems and challenges of the region”.

Although post‐industrial decline is experienced by a multi‐ethnic working‐class, right‐wing populism's racialising frame means that reaction is increasingly articulated through a politics of English nationalist resentment in public discourse, particularly where there is a declining class trajectory (Mann & Fenton, 2017; Virdee & McGeever, 2018). The long‐term impacts of industrial closure in the coalfields are demonstrated by Beynon and Hudson (2021), not only due to the destruction of the economic base, but also in terms of a loss of status within the national economy and loss of culture and identity. Additionally, coalfield communities felt abandoned by the Labour Party and their failure to address gradual decline. As Makin‐Waite (2021) illustrates in relation to Burnley, the former mill town, loss of faith in traditional political affiliations creates a void for populist nationalism to exploit. As such, it follows that resentment is likely to be present in deindustrialising coalfield communities and can be expressed through themes of entitlement to welfare, housing, jobs and health care, as well as “fairness, civility, loss of community, political correctness and the lack of voice” (Mann & Fenton, 2017, p. 39).

In sum, this article contributes to the literature by extending analysis beyond established multi‐ethnic metropolitan localities to consider a much smaller former colliery town, foregrounding the significance of place in our understanding of how immigration is responded to. In doing so, the role of central government funding streams in framing how social problems are perceived is highlighted and the implications this has for localities.

## 3 METHODOLOGY

This research draws on data collected in Shirebrook, a town in the Bolsover district of Derbyshire approximately five miles from Mansfield. The decision to use Shirebrook's real name in any outputs from this research project was due to the presence of much‐publicized sportswear retailer, Sports Direct's warehouse meaning that, even with a pseudonym, identifying Shirebrook would be relatively easy. Coal mining dominated much of Shirebrook's history, triggering its rapid development from a small rural settlement of less than 600 people in 1891 to a town of 11,116 by 1911. By Gilbert's definition, Shirebrook was a *miners*' town, characterized as “tightly‐knit single‐industry communities, socially and often geographically isolated and distinctive” (Gilbert, 1995, p. 51). Widespread deindustrialization from the 1980s directly impacted the town; Shirebrook colliery, which employed 2000 people, closed in 1993, and a substantial number of jobs were lost in the manufacturing and textiles industries in the wider area. Subsequently, the coalfields have been blighted by persistent economic and social problems (Phillips, 2018).

As part of the regeneration work intended to offset the impact of the colliery's closure, Sports Direct relocated their head office and main distribution warehouse to the site of the former colliery. Sports Direct is a sportswear and equipment retailer focusing on the provision of a wide range of products, high levels of stock, and cheap pricing. By 2016 at Sports Direct's Shirebrook base there were around 200 directly employed permanent members of staff, and 3000 temporary agency workers working primarily to dispatch items to Sports Direct's physical stores and for online orders. Sports Direct is typical of the low‐waged and insecure work that has moved into Britain's former industrial areas, which relies disproportionately on the young and migrant labor (Beatty & Fothergill, 2017). An exposé of the working conditions in the Shirebrook warehouse caught the attention of the local and national media (Goodley & Ashby, 2015), leading to a parliamentary select committee investigation into working conditions. Following this, several articles appeared in the national press blaming East European migrants, drawn in to Shirebrook to meet Sports Direct's labor demands, for social problems in the town (Moore & Ramsay, 2017).

Shirebrook's overall population declined between 1981 (11,077) and 2011 (10,885) (Centre for Towns, 2021), rising slightly to a mid‐2017 estimate of 11,750 at the time of fieldwork (ONS, 2020). Average age increased considerably between the 1981 and 2011 censuses: Over 65 s have increased by 28.3%, whilst 16–24‐year‐olds have decreased by 24% and under 16 s by 20.1% (Centre for Towns, 2021). The aging of small towns brings policy challenges for healthcare, transport, housing, and education (Warren, 2018), and is likely to draw in migrant workers to meet any new labor demands. Shirebrook's population was 94.8% white and British in 2011 (Derbyshire Observatory, 2020). Data on EU migration for a relatively small area like Shirebrook are difficult to attain; however, Bolsover had one of the largest increases in non‐UK born residents in the East Midlands between 2001–2011, the majority of which are from Poland (Migration Observatory, 2017). Shirebrook is the most deprived town in Derbyshire, with high levels of child poverty and fares poorly on deprivation indicators for housing, economy, education, and health, and wellbeing (Derbyshire Observatory, 2020). Shirebrook has one of the lowest (pre‐Covid) economic activity rates in Derbyshire (63.7%) yet relatively low unemployment rates due in part to the “hidden” unemployment frequently found in post‐industrial towns (Beatty et al., 2007). Employment in Bolsover is primarily in care, services and elementary occupations, average pay is below the national average, and the East Midlands has one of the highest concentrations of temporary agency workers anywhere in the UK (Ball et al., 2017).

This research draws on data collected as part of a multimethod ethnographic study exploring economic and social change conducted between October 2016 and December 2017. The ethnography included 667 h of participant observation in a variety of public settings including the high street and marketplace, community events, cafes, pubs, library, and council and community group meetings. I also volunteered at two organizations: a welfare rights center that provided advice for people navigating the benefits system, support with appeals and tribunals, and foodbank referrals; and at English for Speakers of Other Languages (ESOL) classes organized by Unite the Union. Research methods included biographical interviews, photo‐elicitation interviews, participant observation and document analysis. This paper focuses primarily on data collected from documentary analysis, particularly the Bolsover Partnership's (2017) *Building Resilience* program: a successful bid for funding from the Department for Communities and Local Government's (DCLG) Controlling Migration Fund (CMF) led by the Bolsover LA. The CMF has its origins in the Labour government's 2009 Migration Impacts Fund, which was scrapped by the coalition government in 2010 and then reintroduced as the CMF in 2016. Its purpose is to help local authorities manage the impact of migration in local communities (MHCLG, 2018). Biographical interviews are also a main source of data for this paper. These were conducted by the author with 27 participants lasting between 45 min and two hours. The sample included 15 men and 12 women aged between 22 and 74; all were white, 17 were born in the UK and 10 were Polish. The biographical focus was designed to capture temporality, including the life trajectory of the participants, trajectory of place and narratives of social change. The focus on trajectories of life and place is pertinent to the analysis because it enables participants to provide accounts which they use to make sense of their present position. Questions were organized around six broad themes: home; family history; work history and economic life; the everyday; Shirebrook; and the future. Participants were recruited through the ethnographic fieldwork. The data were managed and analyzed thematically using NVivo software. Codes were developed flexibly, guided by the literature but also emerged from the data itself. Fieldnotes and transcripts were initially coded in vivo when themes relevant to the theoretical framework were identified. This bookmarked sections of data from which further codes were developed iteratively between theory and data. As analysis progressed codes were reorganized by deleting, combining and renaming.

## 4 FINDINGS

### 4.1 The numbers game

This section examines how migrant numbers are used to construct immigration as a social problem. Analysis foregrounds the Local Strategic Partnership's *Building Resilience* program (2017) and how this is shaped by austerity and government funding. The analysis will then explore the way numbers are used by the LA and how they feature in interview testimonies with residents. According to the program website (Bolsover Partnership, 2021), an increase in migrants settling in Shirebrook intensified pressure on public services and heightened community tensions. This led to a visit from representatives of the DCLG, who invited the Bolsover Partnership to apply for resources from the CMF, which successfully secured £1.26 m of funding in 2017. It is not the intention of this paper to assess the impact of migration on Shirebrook. But the initiative itself, framed by the CMF, played a key role in constructing immigration as a problem before we even look at the narratives within the document. The first issue to consider is austerity and the significant reduction in local authority budgets known to have impacted poor communities, like Shirebrook, particularly hard (Fitzgerald, 2018; Gray & Barford, 2018). These cuts mean that local authorities must act to find alternative funding sources to meet demands. The DCLG invited the Bolsover Partnership to bid for money from the CMF and so local issues *must* then be framed in relation to immigration if they are to access much‐needed financial resources, despite a lack of evidence to suggest immigration increases pressure on resources (Portes, 2019; Thomas, 2019). The role of LAs is politically restricted to the local situation and available funding streams, making it difficult for them to identify social and economic issues originating from national decision making as the source of problems (Jones, 2015, p. 126). This means that the impact on local communities, resources and services potentially emanating from other processes such as deindustrialization and austerity—neither of which feature in *Building Resilience* (Bolsover Partnership, 2017)—are disregarded and immigration persists as the sole contextualization for social issues. Therefore, as “specialists in symbolic production” (Bourdieu, 1991, p. 168), the LA, via a central government funding stream, designate immigration as *the* legitimate social problem effecting Shirebrook, which has the power to shape public perception of migrants and immigration.

The LA's adoption of the concept of *resilience* is also influenced by “political and bureaucratic structures” (Jones, 2015, p. 127). Resilience discourse has come to prominence as a local and central government policy agenda, particularly in the context of austerity and the promotion of voluntarism and localism. There is significant academic debate over the concept (e.g., Dagdeviren & Donoghue, 2019; Hickman, 2018) but broadly, resilience policy involves placing responsibility onto civil society and individual community members and away from the state in the context of hardship, and is increasingly mobilized in relation to immigration and “community cohesion”. Recent work by the anti‐racism group, Hope not Hate, has situated anti‐immigrant sentiment in English and Welsh towns as an issue of community resilience (Clarke et al., 2020). Garratt et al. (2021) note how resilience discourse in relation to community cohesion extends threats to resilience beyond shocks created by capitalism, to particular sections of the population. “Resilience, as an alleged imputed quality of communities implies, above all, their capacity to maintain themselves in the face of change” (Garratt et al., 2021, p. 58). Therefore, by mobilizing resilience in the context of immigration, the *Building Resilience* program constructs migrants and immigration as the threats Shirebrook residents must be resilient in the face of.

The logic of numbers is a key feature in migration discourse and, while the *Building Resilience* program plays the “numbers game” (Fox et al., 2012, p. 686), their account of population change is difficult to make sense of. They claim at the 2011 census that there has been a “significant change” in “Ethnic Minorities being 624”; that there has been a ‘change’ of 477 “in country of birth” across Shirebrook's five wards; and that there have been 684 National Insurance Registrations (Bolsover Partnership, 2017, p. 4). However, there is no indication of the time period over which these changes have happened. Significantly, they state the total population as 13,300, but this is for the electoral division which includes the neighboring village of Pleasley, yet they divide the population between Shirebrook's five wards, effectively adding the population of a whole village to Shirebrook's South East ward (Bolsover Partnership, 2017, pp. 3–4). This gives the impression that Shirebrook's population has grown by 2888 from 10,412 in 2001, rather than the actual increase of 473 to 10,885 in 2011 (Centre for Towns, 2021). This modest increase of 4% is significantly less than the 7% population growth for the UK as a whole (ONS, 2011), and longer‐term census data suggest that Shirebrook's total population has remained relatively consistently around 11,000 over the last 100 years. The issue is not so much about accuracy of the numbers of migrants in Shirebrook, and the lack of clarity in the *Building Resilience* program demonstrates the known difficulties in measuring immigration (Anderson, 2017; Devanney et al., 2021); rather, the issue is about what these numbers represent. Devanney et al. (2021) note local and central governments' overreliance on statistical data in the formation of immigration policy. The logic of numbers appears objective and appeals to common‐sense ideas about place as a container that becomes “full” (Charteris‐Black, 2006). As Anderson (2017, p. 1529) argues, the state is “always open to the charge that there are “too many” migrants”.

Participants' accounts of the impact of immigration on Shirebrook were often ambivalent. The same person could remark on how migrants had contributed to the revitalization of the town but then, through the logic of numbers, argue that there were simply ‘too many’ in Shirebrook. For example, I asked Victoria, a 35‐year‐old unemployed hairdresser, how Shirebrook had changed:

Obviously the ratio of people, or the influx of Eastern Europeans has sort of boosted the population I think really … But I think at the end of the day, Shirebrook was going quite downhill before Sports Direct opened and the Eastern Europeans came over. I mean for example, there's quite a few Polish shops on the marketplace and things like that. But if they hadn't've opened, the marketplace would've been an absolute dive because things were just closing one after another … I just think that has made a difference in the fact that it has kept things open, it's kept thing running in Shirebrook. The income is still coming into Shirebrook instead of going out to wherever … Now I think the population seems to have just rocketed, which is ok but it's then starting to drain the resources … The main problem is the fact of just purely the amount of people, there's just not enough space, I suppose, for that many people.

Victoria's account demonstrates the complex way many residents interpret change in Shirebrook. On the one hand immigration is interpreted as positive in terms of a much‐needed boost to the declining marketplace, but on the other is construed as a drain on resources and the idea that Shirebrook is an already full container that cannot accommodate more people (Charteris‐Black, 2006). Residents' ambivalence is significant because it demonstrates the potential for a positive interpretation of migrant presence and so does not suggest a resolute anti‐immigration stance. However, in the context of strained material circumstances and a sense of competition over few resources, the prevailing interpretation is that there are “too many” immigrants in Shirebrook which is legitimized and sustained by the framing of social problems by the LA via the CMF and heightened anti‐immigrant discourse more broadly. Migrant numbers were also articulated in both policy and interview data using liquid metaphors, known to contribute to othering (Fox et al., 2012):

The *flow* of migrants from EU countries continues via the recruitment agencies employed by Sports Direct. (Bolsover Partnership, 2017, p. 5, emphasis added)

Over the years the Market Square in Shirebrook has become uninviting and tired which has subsequently diminished many opportunities to thrive and has attracted higher Anti Social [sic] Behaviour following the *surge* of migrant workers. (Bolsover Partnership, 2017, p. 22, emphasis added)

When yer live in an area where erm, yer've got an *influx* o'people and yer've lost control over it … it meks yer think it's about time we did summat about this. (Craig, a 47‐year‐old, works in the building trade, emphasis added)

Fox et al.'s (2012, p. 687) analysis of the racialization of Eastern European migration in the tabloid media demonstrates how news stories about migrant numbers “invite concerns and even incite fear”. The use of liquid metaphors exacerbates these concerns and presents immigration as out of control, illustrated by the above quote from Craig. The idea of control is particularly significant and is not simply about loss of control over the movement of people, but is intimately linked to loss of control over social change (Charteris‐Black, 2006). As such, these metaphors evoke memories of a more meaningful past which is, by contrast, disrupted by change for the worse and so resonate with fears associated with a loss of power and status. The idea of control then, is about controlling the ‘negatively evaluated social changes’ (Charteris‐Black, 2006, p. 573) which has come to be represented by migrants via populist nationalism and the CMF's framing of social problems.

### 4.2 Material and symbolic landscapes of place

This section draws on Shirebrook's “in‐betweenness” that sees it represented as both urban and rural. The materiality and esthetics of place will also be explored, demonstrating how the town's perceived decline is used to position immigration as disorderly and out of place. In the opening pages of the *Building Resilience* program Shirebrook is described as one of Bolsover's “most deprived ex‐industrial communities” (Bolsover Partnership, 2017, p. 2). This characterization draws on typologies associated with urban localities to rationalize that immigration into an area already struggling with deprivation and economic change is problematic (Burdsey, 2016). The use of ‘deprived’ alongside ‘ex‐industrial’ also carries symbolic weight, signifying the “left behind”. Even when these terms are used in reference to areas with larger multicultural populations, as demonstrated in Rhodes et al.'s research (Rhodes et al., 2019) in Oldham and Miah et al.'s (2020) in Dewsbury, the constituency imagined as ‘left behind’ is a working‐class that happens to be white. As such, Shirebrook is constructed as a place where at best there are “legitimate” concerns about immigration, and at worse potential volatility toward outsiders (Miah et al., 2020; Thomas et al., 2018). Elsewhere in the document the Local Strategic Partnership switches to instead invoke a sense of rural nostalgia and stable white Englishness disrupted by the arrival of Eastern European migrants:

Bolsover district has seen increases in Eastern Europeans over the last decade. Being a small rural district, with a static population of 75,866 representing a higher percentage of white, UK born British residents (Census 2011) the increase is significant. (Bolsover Partnership, 2017, p. 3)

Jones et al. (2015) illustrates how nationalist narratives of British identity, imagined to be white and unchanging but under threat from emergent multicultural forms of Britishness, is a feature of community cohesion debates. These narratives are particularly effective in relation to the countryside which has a long history as a foundation of English national identity (Bonnett, 1998; Neal, 2002). As such, “[t]he narrative of a countryside invaded by immigrants invokes unspoken connotations of both race and nation” (Jones, 2015, p. 46).

In an interview, Sheila, a 54‐year‐old voluntary worker, drew on Shirebrook's older “pit *village*” identity to invoke an image of a rural location unable to accommodate migrants:

You can't just chuck immigration into a small, it's not a town, it's a village. You can't. Market town? Come on, you can't just chuck that into a small village wi'out no back‐up structure. You can't do that, it's never gonna work, it's never gonna be viable, you will always get these problems. Probably in cities and that you can get away wi' a lot more. But even in cities you've still got your subcultures ant yer, cus you'll go to a part of Sheffield and it's all like Pakistanis and there's a white part of Sheffield, but you can't have those subcultures in little tiny villages wi'out it causing problems.

Sheila stresses that Shirebrook is a village through opposition to the practice of rebranding former coalfield communities as “market towns” after deindustrialization (Bright, 2011). First, she justifies the need for greater immigration controls through the invocation of Shirebrook as a “tiny little village” that lacks the “back‐up structure” to absorb migrants, indicating the already strained material conditions there. Significantly, migration into the town is not only a recent phenomenon. Post‐war European Voluntary Workers moved to Shirebrook and, as a productive colliery, mining families migrated to the town from declining coalfields between the 1960s and 1980s, obtaining council houses in the town's new estates. Relative prosperity and the way collieries invested in the communities they were embedded in at that time (Beynon & Hudson, 2021) meant that any impact from migration was less obviously felt. However, in the context of austerity and disinvestment strained material conditions *are* felt and are interpreted as resulting from immigration when framed that way by the LA and the CMF and in the context of heightened anti‐immigrant sentiment in political and public discourse. Second, Sheila contrasts Shirebrook with existing fragmentation in the city, by focussing on the “village” element of Shirebrook's older “pit village” identity she invokes the image of a tight‐knit traditional community where emergent diversity would inevitably be problematic.

Using other localities as metaphors to make community cohesion intelligible also works though place. Policy practitioners in Jones' (2015) study primarily used metaphors to present a positive image of their own LA by contrasting it with other places known for community cohesion problems. Jones (2015, p. 114) notes how particular place names require no further elaboration to provoke “imagined geographies of cohesion” because of locally and nationally recognized associations between the place and the problem. For example, in the period after the 2001 riots, Oldham and Bradford were frequently named to signify ethnic segregation and tension. In the *Building Resilience* program, place metaphors were used to indicate the type of place Shirebrook is, and the problems faced:

A Community Cohesion Conference was organised by Bolsover Partnership in November 2014 attracting 70+ stakeholders, demonstrating the importance that partners attribute to addressing the issues of migration and the effects on local communities and service providers. Colleagues from Boston Borough Council and Nottingham City Council were invited so partners were able to hear of their experiences and approaches in dealing with the impact of migration on their communities. (Bolsover Partnership, 2017, pp. 6–7)

The reference to Nottingham, one of the closest major cities to Shirebrook, arguably signifies a metropolitan multi‐ethnic urban area with long histories of multiculturalism (e.g., McKenzie, 2015, pp. 31–34). This positions Nottingham City Council as an authoritative figure on community cohesion that the Local Strategic Partnership can learn from, Shirebrook is then imagined in opposition to this: as a non‐urban area negotiating immigration for the first time. Post‐referendum, Boston in Lincolnshire, is arguably a clearer metaphor for cohesion problems. A highly segregated, semi‐rural area with a high concentration and rapid increase of Eastern European migration, Boston received considerable media attention after recording the highest “Leave” vote in the 2016 EU referendum (Lumsden et al., 2019). As such, Boston signifies the type of community cohesion issues faced in Shirebrook though EU migration and the backlash against this represented by Brexit as “white” working‐class rebellion. The Bolsover Partnership then build further on the signified fear of a racist white backlash by commenting that “tensions within Shirebrook … escalated to the point where some members of the resident community demanded action this including [sic] organised marches and demonstrations” (2017, p. 7), and how this caught the attention of the local and national media (2017, pp. 8–12).

The *Building Resilience* program builds on heightened tensions by drawing on the significant media gaze trained on Shirebrook triggered by revelations over working conditions at Sports Direct and stories about migrants' use of public space including claimed anti‐social behavior and street‐drinking attributed to Polish “culture” (Bolsover Partnership, 2017, pp. 6–11). There is a strong emphasis on integration, a term mentioned 18 times in the document, suggesting relationships in Shirebrook are fractious and appealing to broader concerns associated with ethnic segregation and community cohesion (Lentin & Titley, 2011). For example: “The pace and nature of change and the resulting sense of powerlessness contribute to growing levels of resentment and creates barriers to integration and community cohesion” (Bolsover Partnership, 2017, pp. 17–18). This signifies the presence of a migrant population unwilling or unable to integrate with a resentful “white” working‐class. Much of the discussion relates to “differences in culture” and, drawing on media representations, there is a suggestion that street drinking is an undesirable feature of Polish culture that requires intervention (Bolsover Partnership, 2017, pp. 12, 31–33). This is a racialising discourse, which positions Polish culture as inferior and incompatible with the dominant English culture (Patel & Connelly, 2019; Virdee & McGeever, 2018). But this is also reflective of Shirebrook's aging population and the relative differences in ages between settled and new migrant populations. The anxieties associated with anti‐social behavior are embodied by young men, so while overall population numbers have remained relatively static, the ethnic composition by generation has changed.

The concern over the presence of migrants and their use of public space is also articulated through both the materiality and esthetics of place. An increase in the number of Houses of Multiple Occupancy (HMO) to accommodate migrant workers is argued to have contributed to growing community tensions and complaints from Shirebrook residents, initiating an increase in Environmental Health issues, including rodent infestations, domestic noise, housing disrepair and overcrowding, fly‐tipping, and abandoned vehicles (Bolsover Partnership, 2017, pp. 26–28). Migrants' use of Shirebrook marketplace is associated with it becoming “uninviting and tired” (Bolsover Partnership, 2017, p. 22), which is responded to with initiatives aimed at esthetic improvement. First, this includes “painting the shop fronts and buildings in the market square” to support “a vibrant and inviting place where the existing residents will want to visit”, leading to a reduction in anti‐social behavior (Bolsover Partnership, 2017, p. 22). Second, Bolsover Partnership (2017, pp. 30–33) propose to improve migrants' awareness of “social norms” by implementing “Nudge Theory principles”:

Spraying foot prints on the walk ways in the square will direct individuals and groups to key points such as seating and waste facilities to further support the reduction in ASB [Anti‐Social Behaviour] incedents [sic] in the market square. This will lead to a reduction in groups “hanging around” and litter around the square

Smith et al. (2021) illustrate how everyday esthetic judgments are potentially racialising through repeated juxtapositions of the material consequences of poverty, such as squalor and disorder, with the presence of diverse populations. The examples above demonstrate this relationship which is compounded by proposed interventions aimed at establishing integration through orderliness, reinforcing the notion that “perceived disorder or messiness of local space can thus be taken … as aesthetic evidence of … (racialised) disorder” (Smith et al., 2021,p. 105). These dynamics associated with “culture” and the materiality and esthetics of place also featured in interviews:

Their culture as well has ruined this area … Because of what was going on, their way of life, they don't drink indoors their socialising is outside and that's causing trouble … Yer can go anywhere and there's beer cans galore … under a tree or a lamppost and you'll see a pile of cans … They were crapping, all that kind of thing, sex in the bushes, all in a park where children are goin' to school. (Henry, 74‐year‐old, retired postal worker)

We do not drink on our parks, simple as … This is not the Shirebrook way, I do not know if it's a city way but it's not a Shirebrook way. We do not drink on parks, we do not make noises or drink where our elderly are (Sheila)Henry draws on the materiality and esthetics of place through reference to litter, sex, crap and toilet paper ascribed to the faulty culture of Polish migrants said to have “ruined” Shirebrook. Sheila's account works through place differently, marking Polish culture and the ‘city way’ as inferior to the ‘Shirebrook way’, signifying a homogenous imagined community characterized by civility and moral superiority.

The construction of immigration as a social problem in Shirebrook is rooted in the material and symbolic landscapes of place. The significance of Shirebrook as a former mining town, and as such “in‐between” urban and rural localities, is illustrated through these examples. Representations of the town shift dependent on the argument and the imagery wished to be invoked to construct immigration as problematic, whether that is a deprived “left behind” town with the potential for populist backlash, or a hitherto static and tight‐knit rural village with no prior experience of immigration.

### 4.3 Access to declining resources

The final section examines access to resources, in particular the LA's role in defining immigration as the cause for declining services, constructing it as *the* legitimate social problem effecting Shirebrook. The *Building Resilience* program states that immigration has led to:

a strain on public service providers such as the Police with the number of ASB [Anti‐Social Behaviour] Hate Crime Dispersals, the Fire Service with HMO [House of Multiple Occupancy] issues and fires, Community Safety Team, the Local Authorities and Town Councils, HMO Enforcement, litter and detritus, Social Services, School numbers of pupils and Health providers with double appointments. (Bolsover Partnership, 2017, p. 3)

The program focuses significantly on Shirebrook Health Centre, where it is said that “migrants” needs have placed a significant strain on their service and this has impacted upon local residents such as by the increased use of double appointments' (Bolsover Partnership, 2017, p. 36) to allow time for translation for non‐English speakers. The impact immigration has on health services is debated. Devanney et al. (2021) found increased pressure on some GP practices in areas already suffering from the impacts of multiple deprivation, and also noted that healthcare professionals highlighted how the use of interpreters had implications for service delivery. However, other research has found that any impact from immigration on NHS waiting times was limited to immediately after EU accession in 2004 in deprived areas outside of London, and more broadly there is little evidence to suggest that immigration has a negative impact on the NHS (Giuntella et al., 2018; Wadsworth, 2013) or is a burden on welfare in general (Hansen, 2021).

There are alternative explanations for any decline in services at Shirebrook Health Centre, but which remain absent from the *Building Resilience* program. First, as is common amongst villages and small towns across the UK, Shirebrook has an aging population (Centre for Towns, 2021) which has greater healthcare needs putting increased strain on health services (Warren, 2018). Second is Shirebrook's poorer than average health (Derbyshire Observatory, 2021)—a common feature of the former coalfields (Beynon & Hudson, 2021)—and how health inequalities have been exacerbated by austerity and welfare reform (Bambra & Garthwaite, 2015). Austerity has also negatively impacted on real‐term healthcare spending despite its apparent protection from cuts (Fetzer, 2018; Taylor‐Gooby, 2012). Hansen's (2021) research in Sweden demonstrates that migration actually generates increased revenue, rather than the orthodox interpretation of low‐wage migration draining resources. However, this surplus is also stimulated by investing government grants into the demographically aging and depopulating localities where migrants are then directed to, rather than attempting to balance the books through austerity, or by creating a stratified system that denies migrants access to welfare (Hansen, 2021). A key difference is that the investment in Sweden that Hansen (2021, p. 183) highlights is proactive: migration coincides with significant investment which benefits the locality and so is seen as a “blessing” rather than burden. The CMF, however, is reactive where investment is understood as necessary to “fix” problems attributed to migration. The key point being that the DCLG, through the CMF, compels the Local Strategic Partnership to use immigration as the foremost contextualization for any strain on resources. The symbolic power of the state works “to impose a … way of seeing the social world” (Bourdieu, 1991, p. 106), and so constructs immigration as the principal social problem effecting Shirebrook. This framing has the power to (re)produce negative perceptions of immigration and can intensify competition over entitlement to declining resources.

The perception that immigration is a drain on resources was evident in the ethnographic data. In particular, I encountered rumors that Polish migrants received preferential healthcare treatment and that there were particular days of the week dedicated to treating them. Healthcare was also brought up in interviews too, for example Michelle, a 48‐year‐old homemaker, commented:

They [the doctors] have to have two appointments when they see a foreigner so somebody can translate for 'em, and apparently [the doctor] said that just lately it's gonna get to the point that they can't tek no more people on cos they just haven't got the room to do it, so yer back t'infrastructure

Craig also associated immigration with declining resources:

there's just too many of them and that's not their fault … I'd like to know how much extra revenue the town and the district councils have got because these migrant workers must be paying some sort of council tax and rates … I mean the Health Centre asked for £600,000 to extend the building so that they could see more people and they said no

Both of these accounts work through the materiality of place, and the recognition that Shirebrook is poorly resourced and yet the participants rarely associated this with austerity. Craig's comment that any pressure on resources was “not their fault” again demonstrates participants' ambivalence with regard to migrants' presence, and both quotes could be interpreted as recognition of the pressure of a growing population on resources. However, it is worth reiterating that there has only recently been a modest increase in Shirebrook's overall population after several years of decline. Craig also recognizes that migrants contribute through “council tax and rates” but any redistribution from that is not felt in Shirebrook. In the context of austerity, the decline in scope for councils to address local issues is well known (Makin‐Waite, 2021), which is exacerbated by an aging population and the impacts of deindustrialization. There is a very real sense of pressure on declining resources, but this is interpreted as a problem created by immigration in the context of the CMF framing and heightened political and popular anti‐immigrant discourse.

Jobs were also articulated as resources impacted by immigration. For example, Simon, a 51‐year‐old, unemployed due to ill health, argued that migration had impacted wages and availability of work:

Yer've got thousands of Polish people coming in, I mean I like Polish people, they're nice people, but it's gonna grate on people's nerves intit, when they can't get jobs. Here though [in] Shirebrook, there is a racist element, yer 've got to say that there is, although I can understand it … The thing is when you bring people in from like another country, like Poland … it's gonna lower the wage rate for local people

Again, Simon demonstrates an ambivalent response and appears uncomfortable aligning himself with what he recognizes as racialized attitudes but does suggest resentment that immigration is a threat to the material conditions of Shirebrook's British‐born residents (Mann & Fenton, 2017; Patel & Connelly, 2019). Sports Direct is typical of the low‐waged precarious work created in the former coalfields (Beynon & Hudson, 2021) and former industrial areas more broadly (Beatty & Fothergill, 2017). Older male former industrial workers can more easily opt out of the labor market thanks to generous redundancy payments, occupational pensions and industrial injury benefits, but this is more difficult for the young (Beatty & Fothergill, 2017). However, Shirebrook's aging population means there are fewer young people who, along with migrants, are more frequently drawn into precarious labor (Ball et al., 2017). Additionally, low‐wage economies mean “that even two incomes could not compare with wages earned in the mine” (Beynon & Hudson, 2021, p. 231), and so low‐wage precarious work is arguably uneconomical for those with dependents (Shilliam, 2018). As such, migrant workers are drawn in to meet labor demands. Therefore, “the consequence of migration has not been to increase unemployment or drive down wages” (Beynon & Hudson, 2021, p. 230). Rather, migration is an outcome of deindustrialization but is construed as a threat in the context of decline and heightened mainstream anti‐immigrant discourse.

## 5 CONCLUSION

This article has contributed to scholarship on race and migration by extending analysis beyond large metropolitan areas to a place “in‐between” an urban/rural dichotomy through the consideration of encounters with racialized difference in a former colliery town. This is significant because post‐industrial towns have been central to debates over emergent forms of anti‐immigrant populism in recent years. The construction of immigration as a social problem is firmly rooted in the economic context and the impacts of austerity, deindustrialization and economic restructuring. Through the concept of “resilience” the burden of negotiating the shocks of uneven capitalist development is placed on civil society and individual community members, and where social problems are located as emanating from particular populations, such as migrants, who are constructed as a threat to community resilience. Despite the difficulties measuring any impact caused by immigration (Anderson, 2017; Devanney et al., 2021), it is constructed as a threat through the logic of numbers which draw on the idea there are simply “too many” for a place to contain. This is particularly potent in an impoverished context when the suggestion is that migration is out of control, evoking fears over negatively evaluated social change and loss in power and status.

The construction of immigration as a social problem is also rooted in the material and symbolic landscapes of place. Here, the significance of the “in‐betweenness” of isolated former coalfield communities comes to the fore, demonstrated at once though urban invocations of a deprived “left‐behind” town unwilling or unable to accommodate racialized others, and a rural stable white Englishness disrupted by the arrival of Eastern European migrants. The everyday esthetics of place are significant here too, and the racialising frame which constructs messiness and disorder as evidence for the need to intervene in the faulty culture of racialized outsiders. Finally, access to declining resources in the construction of immigration as a problem was articulated by residents through a racialized politics of resentment over rightful access. The decline in availability or quality of services was articulated as emanating from the pressures of immigration and rarely interpreted as resulting from austerity or economic restructuring. This contrasts against the findings described by Hansen (2021, p. 185), where migrants settling in smaller depopulating municipalities “rejuvenate ageing communities and add real resources that support the sustainability of welfare services and the local tax base”. A fundamental difference being that the Swedish government departed from austerity measures and instead, purposely and significantly increased spending in migrant‐receiving municipalities, meaning that immigration was seen as an opportunity for declining communities. However, in the UK and constrained by austerity, the CMF works backward compelling local authorities to frame migration as a burden that needs to be managed in order to receive a cash injection. A key strength of this research is that data are collected at a range of analytical scales and so the role of the LA, framed by central government, in constructing immigration as the legitimate cause of social problems at the expense of alternative explanations is made evident. This paper demonstrates the symbolic power of the state to construct immigration as *the* foremost social problem effecting Shirebrook, and the interplay this framing has with local narratives. Negative evaluations of immigration were not necessarily universal across all of Shirebrook's settled residents, and many of my sample demonstrated ambivalent attitudes revealing a complex combination of both positive and negative readings of population change. However, framing of immigration as the legitimate social problem affecting Shirebrook has the potential to produce, incite or legitimize anti‐immigrant sentiment, directing residents toward nationalist populism and intensifying competition over scarce resources.

## CONFLICT OF INTEREST

No conflict of interest has been declared by the author.

## ETHICS STATEMENT

Ethical approval for this research was granted by the Research Ethics Committee, School of Sociology and Social Policy, University of Nottingham in September 2016.

## Data Availability

The data that support the findings of this study are available from the corresponding author upon reasonable request.

## Endnotes

^1^
The term “red wall” entered the UK political lexicon in 2019 to describe traditional Labour voting seats across the English North and Midlands that were won by the Conservative Party in the 2017 and 2019 general elections.

^2^
European Accession refers to the expansion of the EU in 2004 to include Cyprus, Czechia, Estonia, Hungary, Latvia, Lithuania, Malta, Poland, Slovakia, and Slovenia; and the inclusion of Bulgaria and Romania in 2007.

^3^
The “left behind” is a term that first appeared to make sense of emergent support for far‐right and right‐wing populist parties, such as the British National Party and the UK Independence Party. In particular, it gained traction in relation to the 2016 EU referendum and refers to a predominantly white and English working‐class said to be marginalized by globalization and deindustrialization and alienated by liberal political values, immigration and multiculturalism (Rhodes et al., 2019).
